# Supplementary material for: A whole-animal phenotypic drug screen identifies suppressors of atherogenic lipoproteins
Source: eLife. 2026 Aug 27;14:RP105314. doi: 10.7554/eLife.105314 (PMC13521566; doi:10.7554/eLife.105314)
Supplement: Supplementary file 2 [file elife-105314-code1.zip › JHDL_primary_result/CYPROTERONE/CYPROTERONE_JHDL_8_034_024_2019_06_21_11_38.pdf]

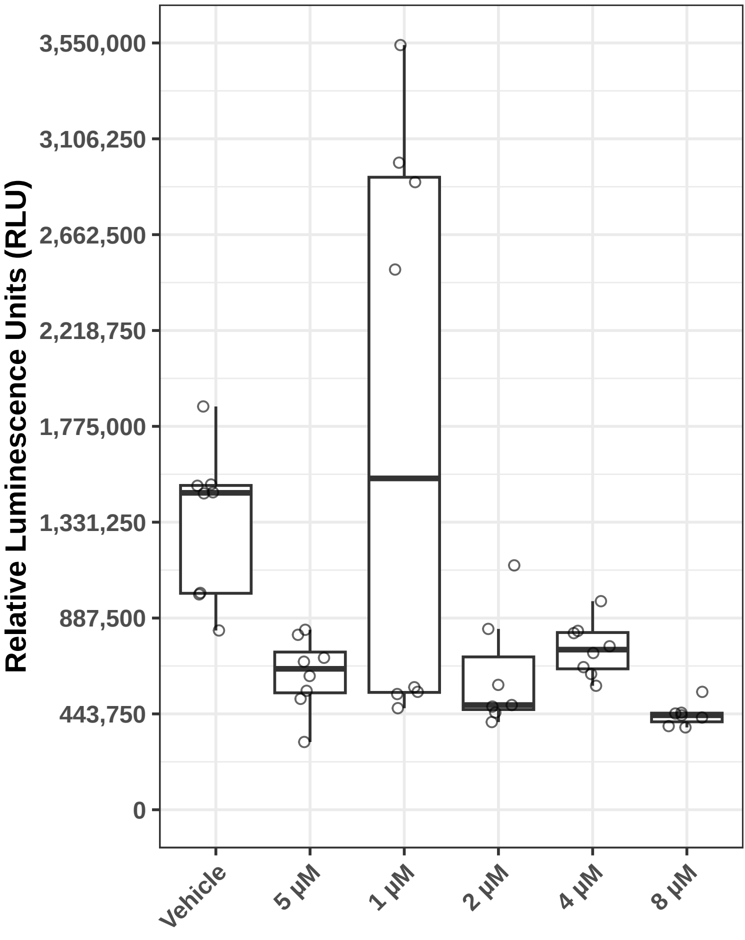

Relative Luminescence Units (RLU)
3,550,000
3,106,250
2,662,500
2,218,750
1,775,000
1,331,250
887,500
443,750
0
Vehicle
5 μM
1 μM
2 μM
4 μM
8 μM
